# Glioma exosomal microRNA-148a-3p promotes tumor angiogenesis through activating the EGFR/MAPK signaling pathway via inhibiting ERRFI1

**DOI:** 10.1186/s12935-020-01566-4

**Published:** 2020-10-27

**Authors:** Meng Wang, Yi Zhao, Zhi-Yun Yu, Ren-De Zhang, Shu-Ang Li, Peng Zhang, Ti-Kun Shan, Xue-You Liu, Ze-Ming Wang, Pei-Chao Zhao, Hong-Wei Sun

**Affiliations:** 1 Department of Neurosurgery, The First Affiliated Hospital of Zhengzhou University, No. 1, Jianshe East RoadHenan Province, Zhengzhou, 450052 Henan Province People’s Republic of China; 2 Department of Translational Medicine Center, The First Affiliated Hospital of Zhengzhou University, Zhengzhou, 450000 People’s Republic of China; 3 Department of Medical, The Third Affiliated Hospital of Zhengzhou University, Zhengzhou, 450052 China; 4 Clinical Systems Biology Laboratories, The First Affiliated Hospital of Zhengzhou University, Zhengzhou, 450000 People’s Republic of China

**Keywords:** Glioma, Angiogenesis, Exosomal microRNA-148a-3p, ERBB receptor feedback inhibitor 1, EGFR/MAPK pathway

## Abstract

**Background:**

Glioma is the most frequent and lethal primary brain malignancy. Amounting evidence has highlighted the importance of exosomal microRNAs (miRNAs or miRs) in this malignancy. This study aimed to investigate the regulatory role of exosomal miR-148a-3p in glioma.

**Methods:**

Bioinformatics analysis was firstly used to predict the target genes of miR-148a-3p. Exosomes were then extracted from normal human astrocytes and glioma cells. Reverse transcription-quantitative polymerase chain reaction (RT-qPCR) was applied to determine the expression patterns of miR-148a-3p and ERBB receptor feedback inhibitor 1 (ERRFI1). Dual-luciferase reporter gene assay was applied to verify the direct binding between miR-148a-3p and ERRFI1. Cell counting kit-8 and tube formation assays were further conducted to assess the proliferation and angiogenic properties of human umbilical vein endothelial cells (HUVECs) in the co-culture system with exosomes. Lastly, glioma tumor models were established in BALB/c nude mice to study the role of exosomal miR-148a-3p in vivo.

**Results:**

miR-148a-3p was highly expressed, while ERRFI1 was poorly expressed in glioma. miR-148a-3p was found to be enriched in glioma cells-derived exosomes and could be transferred to HUVECs via exosomes to promote their proliferation and angiogenesis. ERRFI1 was identified as a target gene of miR-148a-3p. In addition, miR-148a-3p activated the epidermal growth factor receptor (EGFR)/mitogen-activated protein kinase (MAPK) signaling pathway by inhibiting ERRFI1. In the co-culture system, our data demonstrated that glioma cells-derived exosomal miR-148a-3p down-regulated ERRFI1 and activated the EGFR/MAPK signaling pathway, so as to promote cell proliferation and angiogenesis. In vivo experimentation further demonstrated that this mechanism was responsible for the promotive role of exosomal miR-148a-3p in tumorigenesis and angiogenesis.

**Conclusion:**

Taken together, glioma-derived exosomal miR-148a-3p promoted tumor angiogenesis through activation of the EGFR/MAPK signaling pathway by ERRFI1 inhibition.

## Background

Glioma is regarded as a highly-aggressive primary tumor of the brain, whose rapid growth results from glioma-released glutamate, leading to the killing of surrounding excitotoxic cells and rapid tumor invasion [1]. The infiltrative character, complex molecular signaling, localization in the central nervous system, and blood–brain barrier have rendered glioma as one of the most complicated cancers [2]. Interestingly, angiogenesis is also well-known to exert a critical role in the progression of glioma [3]. However, the molecular mechanisms underlying glioma angiogenesis remain unclear, and will be the prime focus of the current study.

Exosomes are small membrane-bound extracellular vesicles (30–100 nm), which are released by numerous types of cells [4]. Recent studies have further shown that exosomes are implicated in the process of glioma progression. For example, exosomes possess the ability to regulate the tumor micro-environment to promote the progression of angiogenesis and tumor development [5]. Evidence has also highlighted the property of exosomes to transport several pro-tumorigenic factors that are associated with the progression of glioblastoma [6]. Additionally, the hard-done work of researchers has also identified that cancer stem cells can potentially release exosomal microRNAs (miRNAs or miRs) to mediate cell–cell communication [7]. Moreover, one such miRNA, miR-148a-3p may play a key role in regulating glioma and angiogenesis. For example, the aberrant expression of miR-148a-3p in glioblastoma cells, and miR-148a in exosomes derived from glioblastoma cells have been proposed to contribute to promotion of cell proliferation and metastasis in glioblastoma (a subtype of glioma) [8]. Furthermore, up-regulated miR-148a expression has also been identified in IDH1R132H human glioblastomas tissues [9]. miR-148a expression is also detected to be higher in glioblastomas tissues than in non-neoplastic brain tissues, and its high levels are associated with poor overall survival in patients with glioblastoma [10]. It has been also demonstrated that miR-148a is dysregulated in medulloblastoma (another subtype of glioma), with its overexpression being correlated with high risk of poor survival [11]. Meanwhile, researchers have identified that glioma cells-derived exosomal miR-9 can be internalized by vascular endothelial cells to promote angiogenesis [12]. Hence, we speculated whether miR-148a could be carried by exosomes of glioma cells to influence the fate of angiogenesis in gliomas.

The ERBB receptor feedback inhibitor 1 (ERRFI1) belongs to the scaffolding adaptor protein family, which plays an important role in the epidermal growth factor receptor (EGFR) signaling pathway [13]. Studies have further indicated that ERRFI1 may be closely related with glioma. One such study has reported that up-regulated ERRFI1 expression is capable of attenuating tumor cell migration [14]. ERRFI1 has also been highlighted to function as a tumor suppressor in glioblastoma by eliminating the malignant potential of glioblastoma cells [15]. In addition, ERRFI1 was reported to have common focal deletions in analysis of 1,057 glioma cases while it could encode Mig6, a feedback inhibitor to block the activation of EGFR [16]. Furthermore, cells expressing high levels of EGFR were previously found to exhibit abnormally low-expressed ERRF1 [17]. Other investigations also indicate that the EGFR/mitogen-activated protein kinase (MAPK) signaling pathway may play a role in glioma. For example, inhibition of EGFR/MAPK signaling pathway has been shown to suppress glioma activation [18]. As Mig6 was shown to be a target of miR-148a-3p [19], the current study set out to investigate the regulatory mechanism of exosomal miR-148a-3p in tumor angiogenesis and glioma progression, which will highlight the further clinical therapeutic strategies to treat glioma.

## Materials and methods

### Ethics statement

All study protocols were in accordance with *the Declaration of Helsinki*, and approved by the Medical and Clinical Research Ethics Committee of the First Affiliated Hospital of Zhengzhou University. Informed written consent was obtained from each participant prior to the study. Animal experimental procedures were in line with the Guide for the Care and Use of Laboratory Animals of the National Institutes of Health. Extensive efforts were made to ensure minimal suffering of the included animals.

### Bioinformatics analysis

Firstly, the Gene Expression Omnibus (GEO) database (https://www.ncbi.nlm.nih.gov/geo/) was used to download the GSE79097 microarray dataset for glioma expression analysis, which is comprised of 3 normal astrocyte samples and 11 primary glioma cell samples. The R language "limma" package was subsequently applied to perform differential analysis, and the false discovery rate (FDR) method was used to calculate the *p* values. The differentially expressed genes were screened with |log2FoldChange|> 2 as the threshold and adj. *p* value < 0.05. Next, the intersection of the downstream target genes of miRNA was obtained through analyses with the miRDB database (https://mirdb.org), mirDIP database (https://ophid.utoronto.ca/mirDIP/index.jsp#r), miRSearch database (https://www.exiqon.com/miRSearch) and TargetScan database. The binding sites between miRNAs and mRNAs were predicted by the TargetScan database (https://www.targetscan.org/vert_71/).

### Clinical samples

Tumor tissues were surgically obtained from 45 patients diagnosed with glioma between January 2017—October 2017 at the First Affiliated Hospital of Zhengzhou University. In addition, 20 patients who underwent intracranial decompression surgery at the First Affiliated Hospital of Zhengzhou University during the same period were selected as the control group. The clinical characteristics of patients and the inclusion criteria are depicted in Additional file 1: Table S1. Bleeding, necrotic and electrocauterized tissues were removed immediately after dissecting and obtaining all samples. The harvested samples were immediately frozen in liquid nitrogen and stored at − 80 °C for further experimentation.

### Cell culture

Human glioma cell lines U-138-MG (U-138, human glioblastoma cell line), U251-MG (U251, human neurogliocytoma cell line), and LN229 (human glioblastoma cell line) as well as human astrocytes (HA), embryonic kidney cell line HEK-293T and human umbilical vein endothelial cells (HUVECs) (Shanghai Institute of Biological Sciences, Chinese Academy of Sciences, Shanghai, China) were tested for short tandem repeats (STR) (Additional file 1: Table S2) and verified to be mycoplasma-free (Sigma-Aldrich, St. Louis, MO, USA). The obtained cells were subsequently cultured in 10% Dulbecco's modified Eagle's medium (DMEM, pH = 7.2, Gibco Company, Grand Island, NY, USA) containing 10% fetal bovine serum (FBS) at 37 °C with 5% CO_2_. The cell growth and morphology were observed and recorded daily, and the medium was renewed every 2 days. Cells were passaged upon reaching 80–85% cell confluence, and subsequent experiments were performed 12 h after cell attachment. None of the cell lines were contaminated with Mycoplasma during the cell culture. Reverse transcription-quantitative polymerase chain reaction (RT-qPCR) was then performed to determine the miR-148a-3p expression patterns in the aforementioned cells. The cell line exhibiting the highest expression of miR-148a-3p was selected for subsequent experimentation.

### Exosome isolation and identification

The exosomes were isolated from normal HA and glioma cells as previously described [20]. In brief, high-quality FBS was ultra-centrifuged at 100,000×*g* and 4 °C for 8 h to remove exosomes in the serum. Next, 5 × 10^6^ cells were placed on a 10 cm culture dish and the medium was discarded when cells reached approximately 80% confluence. After 2 rinses with sterile phosphate-buffered saline (PBS), 10 mL medium containing 10% exosome-depleted FBS was added to the cells, and further cultured in an incubator at 37 °C with 5% CO_2_ for 48 h. The collected culture supernatant was then centrifuged at 500*g* and 4 °C for 15 min to remove the cell debris, followed by another round of centrifugation at 2000*g* and 4 °C for 15 min to remove the apoptotic bodies. The supernatant was further centrifuged at 10,000×*g* and 4 °C for 20 min to remove the large vesicles. The supernatant was then filtered through a 0.22-μm filter, followed by centrifugation at 110,000×*g* and 4 °C for 70 min, re-suspension in sterile PBS and another repeated ultra-centrifugation in successive. Finally, the exosomes were resuspended in 100 μL sterile PBS for the subsequent experimentation.

A total of 20 μL of exosomes was placed on a copper mesh and allow to stand for 3 min. Then 30 μL of phosphotungstic acid solution (pH = 6.8) was added. Exosomes were subsequently counter-stained at room temperature for 5 min, dried under an incandescent lamp, and visualized and photographed using a transmission electron microscope (TEM) at 80 kV. Nanoparticle tracking analyzer (Nanosight, Marlvern, UK) was applied for exosomal concentration and particle size analysis. Identification of exosome specific markers (CD63, CD81 and TSG101) and endoplasmic reticulum (ER) stress-related protein calnexin (CANX) was conducted by means of Western blot analysis. Each experiment was repeated 3 times independently.

### RT-qPCR

The TRIzol reagent (Cat. No. 16096020, Thermo Fisher Scientific, Waltham, MA, USA) was used to extract the total RNA content from tissues or cells. A total of 5 µg total RNA was reverse- transcribed into complementary DNA (cDNA) using a reverse transcription kit (K1622; Fermentas Inc., Ontario, CA, USA). For miRNA detection, MiRcute Plus miRNA First-Strand cDNA Synthesis kit (TIANGEN, Beijing, China) was applied to reverse-transcribe miRNA from the extracted total RNA from tissues or cells. Synthetic exogenous reference cel-miR-39 (1 pmoL/sample; TIANGEN, Beijing, China) was added to the medium (350 μL) or exosomes (100 μg) in advance. miRNAs were extracted from the aforementioned medium or exosomes using mirVana PARIS kits (Ambion, Company, Austin, TX, USA). Real-time qPCR was subsequently performed using the SYBR Premix Ex Taq Reagent kit (Takara Holdings Inc., Kyoto, Japan) and ABI StepOne real-time PCR machine (Applied Biosystems, Carlsbad, CA, USA), while the miRcute Plus miRNA qPCR detection kit (TIANGEN, Beijing, China) was used for qPCR of miRNA. During qPCR, the content of mRNA template was set at 200 ng, while that of miRNA template was 1 μg. For cell and tissue lysates, β-actin was used as the internal reference for mRNA, while U6 was regarded as the internal control for miRNA. In addition, the cel-miR-39 was used as the internal reference for miRNA levels in the medium or exosomes. The primer sequences are listed in Table 1. The 2^−ΔΔCt^ method was used to quantify the relative expression of target genes. ΔΔCt = ΔCt of experimental group—ΔCt of control group.

**Table 1:** Primer sequences for RT-qPCR

Targets	Primer sequence (5′-3′)	
miR-148a-3p	F: GAGACACTCCGACTCTGAGT	R: GTTCTGTAGTGCACTGAC
ERRFI1	F: GGAGCAGTCGCAGTGAGTT	R: GCCTAGAACCCCGTTCACAA
β-actin	F: GCACAGAGCCTCGCCTT	R: GTTGTCGACGACGAGCG
U6	F: CTCGCTTCGGCAGCACA	R: AACGCTTCACGAATTTGCGT
cel-miR-39	F: ACACTCCAGCTGGGTCACCGGGTGTAAATC	R: TGGTGTCGTGGAGTCG

*RT-qPCR* reverse transcription-quantitative polymerase chain reaction, *miR-148a-3p* microRNA-148a-3p, *ERRFI1* ERBB receptor feedback inhibitor 1, *F* forward, *R* reverse

### Western blot analysis

Exosomes were resuspended in pre-chilled lysis buffer (Tris–HCl, 20 mM, pH = 7.5, NaF 10 mM, NaCl 150 mM, 1% Nonidet P-40, phenylmethylsulfonyl fluoride 1 mM and Na3VO4 1 mM) containing a protease inhibitor cocktail (Hoffmann-La Roche Ltd, Basel, Switzerland). The lysate was then dissolved in 3 × Laemmli's sample buffer. Protein samples were boiled for 5 min, separated by 12% sodium dodecyl sulfate–polyacrylamide gel electrophoresis (SDS-PAGE) and transferred onto a nitrocellulose membrane. The membrane was subsequently blocked with 5% non-fat milk in PBS containing 0.5% Tween-20 (PBST) and incubated with primary rabbit antibodies against CD63 (ab68418, dilution ratio of 1: 1000), CD81 (ab109201, dilution ratio of 1: 2000), TSG101 (ab30871, dilution ratio of 1: 1000) and CANX (ab22595, dilution ratio of 1: 1000) at 4 °C overnight. The following day, the nitrocellulose membrane was washed and incubated with horseradish peroxidase-conjugated secondary antibody (ab6721, dilution ratio of 1: 10,000) at room temperature. The nitrocellulose membrane was washed and developed using an enhanced chemiluminescence (ECL) detection system (170–8280, Bio-Rad Laboratories Inc.; Hercules, CA, USA). Ponceau S staining was applied as an internal reference (Additional file 2).

After cells or tissues were lysed, the proteins were isolated and the concentration was quantified using a Bradford assay (Bio-Rad, Inc., Hercules, CA, USA). Proteins were then subjected to 10% SDS-PAGE and transferred to a nitrocellulose membrane. The primary rabbit antibodies included ERRFI1 (ab227944, dilution ratio of 1: 1000), EGFR (ab52894, dilution ratio of 1: 2000), phosphorylated (p)-EGFR (ab40815, dilution ratio of 1: 2000), ERK (ab17942, dilution ratio of 1: 1000), p-ERK (ab201015, dilution ratio of 1: 1000), vascular endothelial growth factor (VEGF) (ab53465, dilution ratio of 1: 1000) and VEGFR2 (ab39638, dilution ratio of 1: 1000) with glyceraldehyde-3-phosphate dehydrogenase (GAPDH) (ab181602, dilution ratio of 1: 10,000) serving as an internal reference. All the aforementioned antibodies were purchased from Abcam (Cambridge, UK). Data were analyzed with the Quantity One v4.6.2 software. The relative protein levels were presented with the gray value of the target protein band/ reference protein band. Each experiment was repeated 3 times independently.

### Co-culture of PKH67-labeled exosomes with HUVECs

After the exosomes were isolated from U251 cells, subsequent procedures were conducted according to the instructions of the PKH67 kit (PKH67GL-1KT, Sigma-Aldrich, St. Louis, MO, USA). Briefly, the exosomes were resuspended in 1 mL Diluent C solution, while 4 μL PKH67 ethanol dye solution was diluted in 1 mL Diluent C solution to prepare a 4 × 10^−6^ M dye solution. Next, 1 mL of exosome suspension was mixed with the dye solution for 5 min. The staining was quenched with the addition of 2 mL of 10% bovine serum albumin (BSA) to PBS (D8537, Sigma-Aldrich). Following that, 1.5 mL of sucrose solution was added to the PKH67-stained exosomes, followed by centrifugation at 100,000×g for 2 h at 2—8 °C. The exosomal pellets were resuspended in PBS and transferred to an Amicon filter column. After adding 9 mL of PBS and 0.75 mL of medium, exosomes were then centrifuged at high-speeds of 2000×*g* for 40 min to reduce the volume to 0.5–1 mL.

HUVECs were routinely cultured and seeded, and the medium was renewed after 48 h. HUVECs were then incubated with PKH67-labeled exosomes or PBS for 24 h. The cells were subsequently fixed with 4% paraformaldehyde at room temperature for 30 min and the nuclei were stained with 4′,6-diamidino-2-phenylindole (DAPI) (36308ES11, Yeasen Company, Shanghai, China) for 5 min. Next, the cells were visualized and photographed under a laser scanning confocal microscope (LSCM) (DMi8, Leica, Wetzlar, Germany). HUVECs were then treated with PBS or co-cultured with exosomes isolated from U251 cells, from inhibitor negative control (NC)-transfected U251 cells or from miR-148a-3p inhibitor-transfected U251 cells. Additionally, the HUVECs transfected with overexpression (oe)-NC or oe-ERRFI1 plasmid were treated with PBS or co-cultured with U251 cells-derived exosomes. Each experiment was repeated 3 times independently.

### Cell transfection

Cells were seeded in a 6-well plate at a density of 2 × 10^5^ cells/well 24 h prior to transfection. Transfection was performed according to the protocols of Lipofectamine 2000 reagents (11668019, Thermo Fisher Scientific, Waltham, MA, USA). Initially, 4 μg expression plasmid or 100 μM miRNA inhibitor and 8 μL liposome at the ratio of 1:2 were added into the cells, and uniformly mixed, followed by incubation with 250 μL Opti-MEM at room temperature for 15 min. Then, the cells were cultured with 10% antibiotic-free DMEM and incubated for 48 h before subsequent experiments. The cells were subsequently transfected with miR-148a-3p inhibitor (chemically modified inhibitor specifically targeting miR-148a-3p), miR-148a-3p mimic (chemically modified short dsDNA simulating the function of endogenous miR-148a-3p), oe-ERRFI1 plasmid and the matched NCs (inhibitor NC, mimic NC, oe-NC plasmid) alone or in combination, respectively. All the aforementioned mimic, inhibitor and plasmids were constructed by Shanghai Genechem Co., Ltd. (Shanghai, China).

### Cell counting kit-8 (CCK-8) assay

Cell proliferation was measured using CCK-8 kits (CA1210-100, Beijing Solarbio Science & Technology Co., Ltd., Beijing, China). Cells at the logarithmic phase of growth were seeded in a 96-well plate at a density of 5 × 10^3^ cells per well, and cultured for 3 days. Each well was supplemented with 10 μL of CCK-8 solution and placed in an incubator for 2 h. Subsequently, the absorbance values at 450 nm were measured with a microplate reader (BIO-RAD 680, Bio-Rad, Hercules, CA, US) and the optical density (OD) values were recorded at the 24 h, 48 h and 72 h time intervals, respectively, after which a cell growth curve was plotted.

### Tube formation assay

Matrigel (354234, Shanghai Shanran Biotechnology Co., Ltd., Shanghai, China) was placed in a refrigerator at 4 °C overnight prior to the experiments. The Matrigel was placed in a wet-box after melting and solidification in an incubator at 37 °C for 30 min. After transfection or treatment with exosomes for 24 h, the complete culture medium containing FBS was replaced with serum-free medium for 1-h starvation. After that, the harvested cells were then resuspended in Roswell Park Memorial Institute (RPMI)-1640 medium to prepare a cell suspension with a density of 2 × 10^5^ cells/mL. At last, 50 μL of cell suspension was seeded in the gel-coated slides at a density of 1 × 10^4^ cells, with each test conducted in triplicate. After incubation for 12 h, the cells were photographed using a Leica inverted phase contrast microscope (Leica DMi1, Leica Microsystems Trading Co., Ltd., Shanghai). The length of capillary lumen formed in cells was calculated in at least 3 fields from each group with the mage-Pro Plus (version 6.0) under 100× magnification. Each experiment was repeated three times independently.

### Dual-luciferase reporter assay

After site-directed mutagenesis in core sequence (5′-TGCACTGA-3′) of the ERRFI1 mRNA 3′-untranslated regions (3′-UTR) where a putative miR-148a-3p binding site existed, the ERRFI1-3′-UTR-mutant (MUT) sequence was generated. The pmiR-RB-REPORT plasmid (RiboBio Co., Ltd., Guangzhou, China) was subsequently cleaved with restriction enzymes and then, the target sequence of the artificially synthesized wild type (WT) and MUT were inserted into the pmiR-RB-REPORT vector (RiboBio Co., Ltd., Guangzhou, China), respectively. The recombinant WT and MUT luciferase reporter plasmids were sequenced prior to transfection. The vectors containing MUT and WT were co-transfected with mimic-NC or miR-148a-3p mimic into HEK293T cells, respectively. After 48 h of transfection, the cells were collected, lysed and centrifuged for 3–5 min to collect the supernatant. A Renilla luciferase detection kit (YDJ2714, Shanghai Yuduo Biotechnology Co., Ltd., Shanghai, China) was applied to determine the relative luciferase units (RLU), where firefly luciferase was regarded as an internal control. A dual-luciferase reporter analysis system (Promega Co, Madison, WI, USA) was employed for data analysis. Each experiment was repeated 3 times independently.

### Tumor xenograft in nude mice

A total of 40 male Balb/c nude mice (aged 4–6 weeks, weighing 16–22 g) (J004, Nanjing Junke Biotechnology Engineering Co., Ltd., Nanjing, China) with an average age of 5.05 ± 0.78 weeks and average weight of 19.25 ± 1.08 g were selected with 10 mice per group. The U251 cells were detached with 0.25% trypsin, resuspended in sterile PBS and centrifuged several times to remove the FBS from the cell suspension. Next, 5 × 10^6^ cell suspension was injected into the right back scapular region of the nude mice. miR-148a-3p antagomir (chemically inhibit the expression of endogenous miR-148a-3p), miR-148a-3p agomir (chemically mimic the expression of endogenous miR-148a-3p) (80 mg/kg/d, 3 consecutive days) or U251-exo (100 nM/100 µL/mice), and the PBS of equal amounts was delivered into mice via intravenous tail injections. After 3 weeks, the mice were euthanized with intraperitoneal injections of 9% pentobarbital sodium (P3761, SIGMA, St. Louis, USA), followed by the isolation of tumors. The short diameter (a) and long diameter (b) of the tumor were measured with a Vernier caliper, and the tumor volume was calculated according to the formula π (a^2^b)/6, while the tumors were weighed with a balance. Serum samples were collected from mouse peripheral blood, and exosomes were extracted to detect the miR-148a-3p expression patterns (cel-miR-39 was used as internal reference) using RT-qPCR. Tumor tissues were fixed with 10% formaldehyde, dehydrated, embedded with paraffin and then sectioned into 4-μm tissue sections for subsequent experimentation.

### Microvascular density (MVD) detection

CD31 expression was detected using immunohistochemistry (IHC). CD31 expression was visualized under a microscope to determine the MVD, with the Weidner method applied to quantify MVD. Briefly, all fields of view were observed under a low magnification (×100) microscope. Subsequently, 3 areas with the highest density of brownish yellow staining were selected, which were regarded as the “hot spots” of enriched blood vessels. Lastly, the number of microvessels was counted under high magnification. Single or clustered endothelial cells stained with brownish yellow (independent from adjacent microvessels, tumor cells or other connective tissues) were counted as 1 microvessel. At least 3 fields were randomly selected in each group and the number of microvessels was quantified in these fields. The mean value was used to present the MVD.

### Enzyme-linked immunosorbent assay (ELISA)

After HUVECs were treated with exosomes for 24 h, the cell supernatant was collected. Serum separation was performed after 3 weeks of treatment in nude mice. Vascular endothelial growth factor (VEGF) and VEGFR2 were measured according to the instructions of respective ELISA kits (A106111-48T, Shanghai Fusheng Industrial Co., Ltd., Shanghai, China). A Multiskan Spectrum microplate reader (BS-1101, Deutsche Texel Experimental Equipment Co., Ltd., Nanjing, China) was used to record the OD values at a wavelength of 450 nm. A blank control group was set for each test.

### Statistical analysis

SPSS 23.0 statistical software (IBM Corp. Armonk, NY, USA) was used for statistical analyses. Measurement data were presented as mean ± standard error of the mean (SEM). Each experiment was run at least 3 times with parallel wells and control wells set for each group. Two groups of data conforming to normal distribution were compared using unpaired *t* test. Data among multiple groups were compared using one-way analysis of variance (ANOVA), with Tukey's test for data obeying homogeneity of variance, otherwise Dunnett’s test was conducted. Data at different time points among multiple groups were analyzed using repeated measures ANOVA. Pairwise comparisons within groups were performed using Tukey's test. The correlation of two indicators was analyzed using Pearson’s correlation coefficient. A value of *p* < 0.05 indicated statistical significance.

## Results

### miR-148a-3p is enriched in glioma cells-derived exosomes

**Fig. 1:**
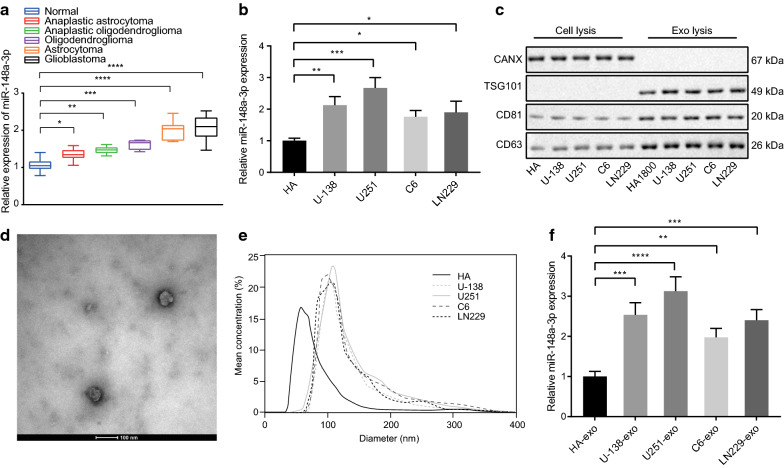
miR-148a-3p is highly expressed in glioma tissues, cell lines and glioma cells-derived exosomes. **a** The box plot showing the miR-148a-3p expression determined by RT-qPCR in non-neoplastic brain tissues of control patients (n = 20) and neoplastic brain tissues of glioma patients (n = 45) including anaplastic astrocytoma, anaplastic oligodendroglioma, oligodendroglioma, astrocytoma, and glioblastoma (U6 was used as internal control). **b** miR-148a-3p expression determined by RT-qPCR in normal HA and glioma cell lines U-138-MG (U-138), U251-MG (U251), and LN229 (U6 was used as internal control). **c** Western blot analysis of exosome specific markers (CD63, CD81, and TSG101), and ER stress-related protein CANX in the exosomes extracted from normal HA and glioma cell lines U-138-MG (U-138), U251-MG (U251), and LN229. **d** Transmission electron microscopic identification of exosomes (scale bar = 100 nm). **e** NanoSight particle size analysis to quantify exosome concentration and average diameter. **f** Expression of miR-148a-3p was determined by RT-qPCR in the exosomes extracted from normal HA and glioma cell lines U-138-MG, U251-MG, and LN229 (U6 was used as internal control). * *p* < 0.05, ** *p* < 0.01, *** *p* < 0.001, and **** *p* < 0.0001 vs. control brain tissues, HAs or HAs-derived exosomes (HA-exo). Data are shown as mean ± SEM. The cell experiments were performed in 3 repeats and each repeat was performed in technical replicate (triplicate). One-way ANOVA was used for data analysis among multiple groups, followed by Tukey's test

Previous literature showed that miR-148a-3p was highly-expressed in glioma tissues and cells [19], and its dysregulation was correlated with the histological grade of glioma [21]. Furthermore, the exosomes derived from glioma cells also possess the ability to deliver miR-148a to promote the occurrence and development of glioma [8]. In the current study, we collected brain tissue samples from 20 patients who underwent intracranial decompression surgery (serving as the control) and 45 patients with various types of gliomas (astrocytoma, oligodendroglioma, anaplastic astrocytoma, anaplastic oligodendrogliomas, and glioblastoma), and then performed RT-qPCR to characterize the miR-148a-3p expression patterns in glioma tissues. The results showed that the expression of miR-148a-3p was higher in tumor tissues of patients with anaplastic astrocytoma, anaplastic oligodendrogliomas, oligodendroglioma, astrocytoma, and glioblastoma relative to that of the non-neoplastic brain tissues, with the highest expression exhibited in glioblastoma tissue samples (Fig. 1a). Subsequently, three human glioma cell lines, U-138-MG, U251-MG, and LN229, and human primary astrocytes HA were selected and cultured, wherein the expression of miR-148a-3p was determined by RT-qPCR. Compared with HA, miR-148a-3p expression was found to be higher in all the glioma cell lines, among which the highest miR-148a-3p expression was found in U251-MG cells (*p* < 0.05, *p* < 0.01, *p* < 0.001) (Fig. 1b), and therefore U251-MG was selected for subsequent experiments. The exosomes in HA, U-138-MG, U251-MG, and LN229 cells were extracted and the expression patterns of exosome-specific marker, CD63, CD81 and TSG101, and ER stress-related protein, CANX were analyzed by Western blot analysis. The data showed that the extracted exosomes were positive for CD63, CD81 and TSG101, but negative for CANX protein (Fig. 1c). Transmission electron microscopy was applied to identify the structure of exosomes, which demonstrated that most exosomes were round- or oval-shaped, with a diameter of approximately 30—150 nm (Fig. 1d). Further analysis of the size and diameter of exosomes using NanoSight particle size analysis revealed that a large proportion of them primarily ranged from 20 to 200 nm in diameter (Fig. 1e). Finally, RT-qPCR was performed to determine the expression patterns of miR-148a-3p in the exosomes derived from different cell lines. The results showed that miR-148a-3p expression was significantly higher in the glioma-derived exosomes in comparison to HA-derived exosomes. Among them, the highest expression of miR-148a-3p was found in U251 cells-derived exosomes (*p* < 0.01, *p* < 0.001, *p* < 0.0001), so U251 cells-derived exosomes was chosen for subsequent experiments (Fig. 1f). Taken together, our data demonstrated that miR-148a-3p was enriched in glioma tissues and cells, as well as glioma cells-derived exosomes.

### Glioma cells-derived exosomal miR-148a-3p promotes vascular endothelial cell proliferation and angiogenesis

**Fig. 2:**
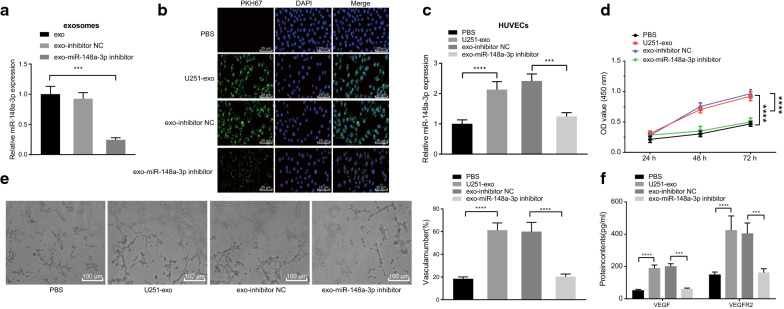
Glioma cells-derived exosomal miR-148a-3p promotes HUVEC proliferation and tube formation. **a** miR-148a-3p expression was determined by RT-qPCR in the exosomes isolated from U251 cells or exosomes from U251 cells transfected with miR-148a-3p inhibitor (U6 was used as internal control). **b** Uptake of PKH67-labeled U251-derived exosomes by HUVECs visualized under LSCM (scale bar = 25 µm). **c** miR-148a-3p expression was determined by RT-qPCR in the HUVECs after co-culture with exosomes from U251 cells transfected with miR-148a-3p inhibitor (U6 was used as internal control). **d** CCK-8 detection of HUVEC proliferation in the co-culture system of HUVECs and exosomes from U251 cells transfected with miR-148a-3p inhibitor. **e** Lumen formation ability of HUVECs in the co-culture system of HUVECs and exosomes from U251 cells transfected with miR-148a-3p inhibitor (scale bar = 100 µm). **f** ELISA detection of VEGF and VEGFR2 expression in cell supernatant in the co-culture system of HUVECs and exosomes from U251 cells transfected with miR-148a-3p inhibitor. * *p* < 0.05, ** *p* < 0.01, *** *p* < 0.001, and **** *p* < 0.0001. U251 mentioned in this figure refers to U251-MG. Data are shown as mean ± SEM. The cell experiments were performed in 3 repeats and each repeat was performed in technical replicate (triplicate). The unpaired *t* test was used for comparison between two groups. The repeated measures ANOVA was used for data analysis at different time points. Comparisons among multiple groups were conducted using one-way ANOVA with Tukey's test

In order to investigate the characteristics and molecular functions of miR-148a-3p carried by exosomes, HUVECs were co-incubated with PBS, the isolated exosomes from U251 cell line or with the exosomes isolated from miR-148a-3p inhibitor-transfected U251 cells, after which RT-qPCR was applied to analyze the expression patterns of miR-148a-3p in the exosomes. The results showed that compared with the exosomes isolated from U251 cells, the expression of miR-148a-3p was increased in the exosomes isolated from the U251 cells transfected with miR-148a-3p inhibitor (*p* < 0.001) (Fig. 2a). The exosomes were labeled with PKH67 and further incubated with HUVECs, and the nuclei exhibited blue coloration, and the exosomes labeled with PKH67 exhibited green fluorescence. Green fluorescence was visualized in the cytoplasm of HUVECs co-cultured with U251 cells-derived exosomes, indicating that the exosomes were successfully internalized by the HUVECs (*p* < 0.05) (Fig. 2b). Moreover, the results of RT-qPCR indicated that the expression of miR-148a-3p was elevated in HUVECs co-cultured with exosomes relative to PBS treatment. Compared with HUVECs co-cultured with exosomes from inhibitor NC-transfected U251 cells, miR-148a-3p expression was found to be decreased in HUVECs co-cultured with exosomes from the miR-148a-3p inhibitor-transfected U251 cells (*p* < 0.001, *p* < 0.0001) (Fig. 2c). These results indicated that U251 cells-derived exosomes delivered miR-148a-3p to HUVECs.

Next, CCK-8 and tube formation assays were performed, and the results demonstrated that HUVEC proliferation and angiogenesis were both enhanced following co-culture with exosomes when compared with PBS treatment. However, HUVEC proliferation and angiogenesis were found to be attenuated in the co-culture system with exosomes from miR-148a-3p inhibitor-transfected U251 cells (*p* < 0.0001) (Fig. 2D, e). In addition, the expression patterns of angiogenesis-related factors, VEGF and VEGFR2, in the cell supernatant were detected by ELISA, which showed that both the expressions of VEGF and VEGFR2 were higher in HUVECs co-cultured with exosomes. On the contrary, deficiency of miR-148a-3p brought about inhibited expressions of VEGF and VEGFR2 that were induced by exosomes (*p* < 0.001, *p* < 0.0001) (Fig. 2F). In summary, these findings indicated that glioma cell U251 promoted HUVEC proliferation and angiogenesis through the delivery of miR-148a-3p via exosomes.

### miR-148a-3p promotes vascular endothelial cell proliferation and angiogenesis

**Fig. 3:**
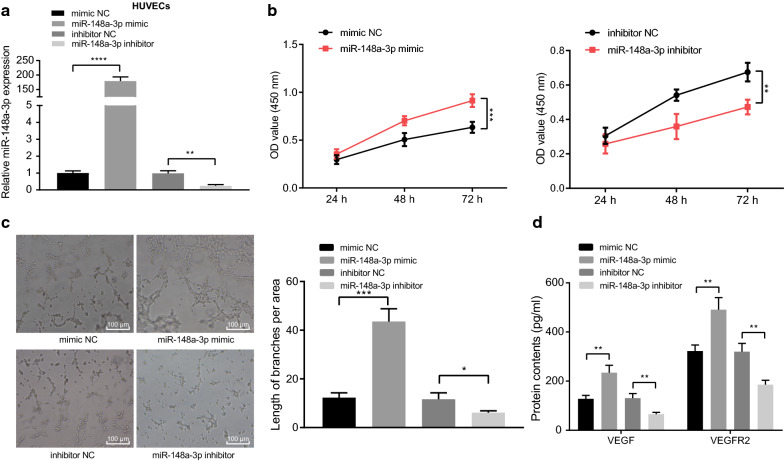
HUVEC proliferation and angiogenesis could be enhanced by miR-148a-3p. **a** Expression of miR-148a-3p was determined by RT-qPCR in HUVECs transiently transfected with miR-148a-3p mimic or inhibitor (U6 was used as internal control). **b** CCK-8 assay to detect proliferation of HUVECs transiently transfected with miR-148a-3p mimic or inhibitor. **c** Tube formation assay to test the angiogenesis ability of HUVECs transiently transfected with miR-148a-3p mimic or inhibitor (scale bar = 100 µm). **d** Expression of VEGF and VEGFR2 was tested using ELISA in cell supernatant upon transient transfection with miR-148a-3p mimic or inhibitor. * *p* < 0.05, ** *p* < 0.01, *** *p* < 0.001, and **** *p* < 0.0001 *vs.* the cells transfected with mimic NC or with inhibitor NC. Data are shown as mean ± SEM. The cell experiments were performed in 3 repeats and each repeat was performed in technical replicate (triplicate). Repeated measures ANOVA was used for data analysis among multiple groups and multiple time points. Unpaired *t* test was used for comparison between the two groups

After uncovering that glioma cell U251 promoted HUVEC proliferation and angiogenesis through exosomal miR-148a-3p, we hypothesized that miR-148a-3p was one of the important factors contributing to HUVEC proliferation and angiogenesis. Subsequently, HUVECs were transfected with miR-148a-3p mimic and inhibitor and their corresponding NCs, after which miR-148a-3p expression patterns were determined by RT-qPCR. The expression of miR-148a-3p in the cells transfected with miR-148a-3p mimic was found to be significantly higher than that in the cells transfected with mimic NC. However, miR-148a-3p expression in the cells transfected with miR-148a-3p inhibitor was significantly decreased (*p* < 0.001, *p* < 0.0001) (Fig. 3a). Meanwhile, CCK-8 and tube formation assays revealed that overexpression of miR-148a-3p promoted HUVEC proliferation and angiogenesis, while depletion of miR-148a-3p inhibited the proliferation and angiogenesis (*p* < 0.05, *p* < 0.01, *p* < 0.0001) (Fig. 3b, c). Based on ELISA results, the expressions of angiogenesis-related factors, VEGF and VEGFR2, were both significantly elevated following miR-148a-3p overexpression. Compared with the cells transfected with inhibitor NC, the expressions of VEGF and VEGFR2 were significantly decreased upon treatment with miR-148a-3p inhibitor (*p* < 0.01) (Fig. 3d). In summary, the obtained findings suggested that miR-148a-3p overexpression promoted HUVEC proliferation and angiogenesis.

### miR-148a-3p activates the EGFR/MAPK signaling pathway by inhibiting ERRFI1

**Fig. 4:**
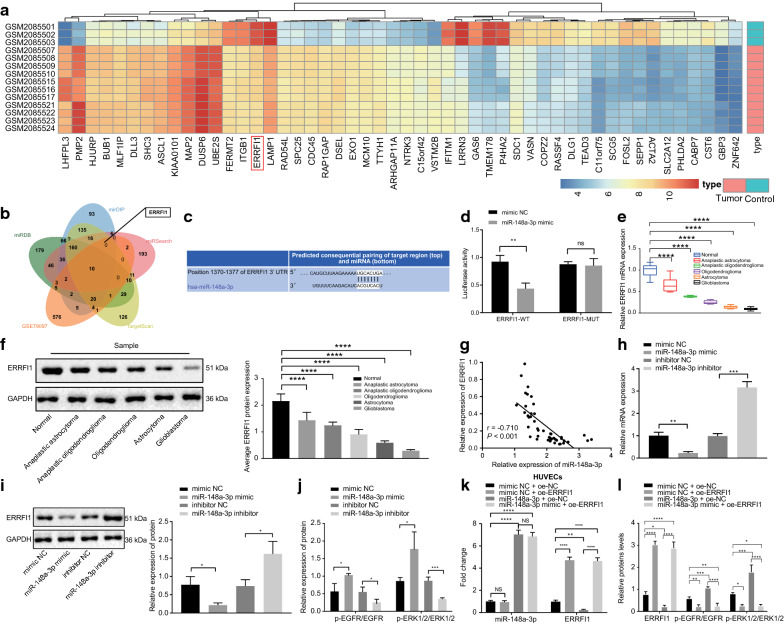
miR-148a-3p activates the EGFR/MAPK signaling pathway through inhibiting ERRFI1 expression. **a** Heat maps of target genes that down-regulated in GSE79097. The abscissa represents the sample number, the ordinate represents the gene names, the tree diagram on the left represents the clustering of gene expression, and the box plot on the upper right represents the scale. **b** Intersection of the miR-148a-3p target genes predicted by miRDB, miDIP, miRSearch, and TargetScan databases with the down-regulated genes in the GSE79097 microarray. The five ellipses in the Figure represent the number of predicted target genes from the four databases and the number of down-regulated genes in the GSE79097 microarray and the intersection represents intersected genes in five sets of data. **c** The binding sites between ERRFI1 and miR-148a-3p were predicted by the TargetScan database (https://www.targetscan.org/vert_71/). **d** Dual-luciferase reporter gene assay validation on the relationship between ERRFI1 and miR-148a-3p in HEK293T cells. **e** Expression of ERRFI1 was determined by RT-qPCR in non-neoplastic brain tissues of control group (n = 20), and neoplastic brain tissues of glioma patients (n = 45) including anaplastic astrocytoma, anaplastic oligodendroglioma, oligodendroglioma, astrocytoma, and glioblastoma (GAPDH was used as internal control). **f** Western blot analysis to detect ERRFI1 expression in the non-neoplastic brain tissues of control group (n = 20), and neoplastic brain tissues of glioma patients (n = 45) including anaplastic astrocytoma, anaplastic oligodendroglioma, oligodendroglioma, astrocytoma, and glioblastoma (GAPDH was used as internal control). **g** Pearson correlation analysis of ERRFI1 expression and miR-148a-3p expression in tumor tissues (n = 45). **h** Expression of ERRFI1 was determined by RT-qPCR in HUVECs transiently transfected with miR-148a-3p mimic or inhibitor (GAPDH was used as internal control). **i** Western blot analysis to analyze ERRFI1 expression in HUVECs transiently transfected with miR-148a-3p mimic or inhibitor. **j** Western blot analysis to detect ratios of p-EGFR/EGFR and p-ERK1/2/ERK1/2 in HUVECs transiently transfected with miR-148a-3p mimic or inhibitor. **k** Expression of miR-148a-3p and ERRFI1 was determined by RT-qPCR in HUVECs transfected with oe-ERRFI1/oe-NC and miR-148a-3p mimic/mimic NC (GAPDH was used as internal control). **l** Western blot analysis of ERRFI1, extent of EGFR and ERK1/2 phosphorylation, and ratios of p-EGFR/EGFR and p-ERK1/2/ERK1/2 in HUVECs transfected with oe-ERRFI1/oe-NC and miR-148a-3p mimic/mimic NC. * *p* < 0.05, ** *p* < 0.01, *** *p* < 0.001, and **** *p* < 0.0001. NS indicated no significant difference. Data are shown as mean ± SEM. The cell experiments were performed in 3 repeats and each repeat was performed in technical replicate (triplicate). Unpaired *t* test was used for comparison between the two groups

To predict the downstream target genes of miR-148a-3p, bioinformatics analysis using miRDB and other databases was pursued. In addition, the glioma expression microarray GSE79097 dataset was obtained from GEO dataset. Differential analysis of both normal and glioma samples in this microarray was performed, which showed that 631 genes were down-regulated in gliomas. The heat map of 50 genes with largest fold changes among the aforementioned down-regulated genes showed that ERRFI1 expression was down-regulated in gliomas (Fig. 4a). Subsequently, the putative target genes of miR-148a-3p were intersected with the down-regulated genes (Fig. 4b), by which 10 candidate target genes were identified, including ERRFI1 (Table 2). Through Targetscan website analysis, it was found that miR-148a-3p and ERRFI1 possessed a potential binding site (Fig. 4c). Hence, ERRFI1 was speculated to be a target of miR-148a-3p. Next, dual-luciferase reporter assay was applied to verify their binding relationship, which showed that the luciferase activity of ERRFI1-WT was inhibited in cells transfected with miR-148a-3p mimic (*p* < 0.01), while that of ERRFI1-MUT was not affected (*p* > 0.05) (Fig. 4d), demonstrating that ERRFI1 was indeed a target gene of miR-148a-3p. Furthermore, RT-qPCR and Western blot analysis were conducted to measure the expression patterns of ERRFI1 in glioma tissues, which revealed that the expression of ERRFI1 was lower in tissue samples from patients with anaplastic astrocytoma, anaplastic oligodendrogliomas, oligodendroglioma, astrocytoma, and glioblastoma than that in the non-neoplastic brain tissue samples (*p* < 0.0001) (Fig. 4e, f). Pearson’s correlation coefficient further showed that ERRFI1 expression was negatively correlated with the miR-148a-3p expression (Fig. 4g). The above results and existing data indicated that ERRFI1 was poorly expressed in gliomas, and was a target of miR-148a-3p. Moreover, miR-148a-3p could suppress the expression of ERRFI1.

**Table 2:** Differential expression of 10 intersection genes in the GSE79097 microarray

Symbol	logFoldChange	*p* value	adj.*p*.Val
IGF1	− 3.244636673	1.55E−19	6.72E−17
MDFIC	− 3.105624786	3.43E−18	9.46E−16
NRP1	− 2.014977195	1.41E−15	1.65E−13
ERRFI1	− 2.71043279	1.61E−11	4.56E−10
CABP7	− 2.344443128	2.39E−11	6.31E−10
SIK1	− 2.518773064	2.86E−09	3.98E−08
INHBB	− 3.834802576	3.19E−09	4.39E−08
COL4A1	− 3.099068154	5.01E−06	2.80E−05
NHS	− 2.707093253	1.00E−05	5.14E−05
EIF4E3	− 2.095916338	1.69E−05	8.10E−05

*IGF1* Insulin-like growth factor 1, *MDFIC* MyoD family inhibitor domain containing, *NRP1* Neuropilin 1, *ERRFI1* ERBB receptor feedback inhibitor 1, *CABP7* calcium-binding protein 7, *SIK1* salt-inducible kinase 1, *INHBB* inhibin beta b, *COL4A1* collagen type IV alpha 1 chain, *NHS* Nance-Horan syndrome, *EIF4E3* eukaryotic translation initiation factor 4E family member 3

Furthermore, HUVECs were transfected with miR-148a-3p mimic, miR-148a-3p inhibitor and their corresponding NCs. The results of RT-qPCR and Western blot analysis showed that overexpression of miR-148a-3p inhibited the ERRFI1 expression, while inhibiting miR-148a-3p promoted the ERRFI1 expression in HUVECs (*p* < 0.05, *p* < 0.01, *p* < 0.001) (Fig. 4h, i). In addition, Western blot analysis demonstrated that overexpression of miR-148a-3p promoted the extents of EGFR and ERK1/2 phosphorylation and the ratios of p-EGFR/EGFR and p-ERK1/2/ERK1/2, while inhibiting miR-148a-3p resulted in opposite reductions (*p* < 0.05, *p* < 0.001) (Fig. 4J). Thereafter, HUVECs were further transfected with oe-ERRFI1/oe-NC and miR-148a-3p mimic/mimic NC in combination. ERRFI1 overexpression led to no alteration in the miR-148a-3p expression (*p* > 0.05), but reduced the extents of EGFR and ERK1/2 phosphorylation and ratios of p-EGFR/EGFR and p-ERK1/2/ERK1/2 (*p* < 0.01, *p* < 0.0001). Heightened miR-148a-3p expression was also noted to diminish the ERRFI1 expression. Concomitant restoration of ERRFI1 reversed the promotive effect of miR-148a-3p on the extents of EGFR and ERK1/2 phosphorylation as well as the ratios of p-EGFR/EGFR and p-ERK1/2/ERK1/2 in HUVECs (*p* < 0.05, *p* < 0.01, *p* < 0.0001) (Fig. 4k, l). These results together indicated that miR-148a-3p activated the EGFR/MAPK signaling pathway by suppressing the ERRFI1 expression.

### Exosomal miR-148a-3p down-regulates the ERRFI1 expression in HUVECs to activate the EGFR/MAPK signaling pathway and promote HUVEC proliferation and angiogenesis

**Fig. 5:**
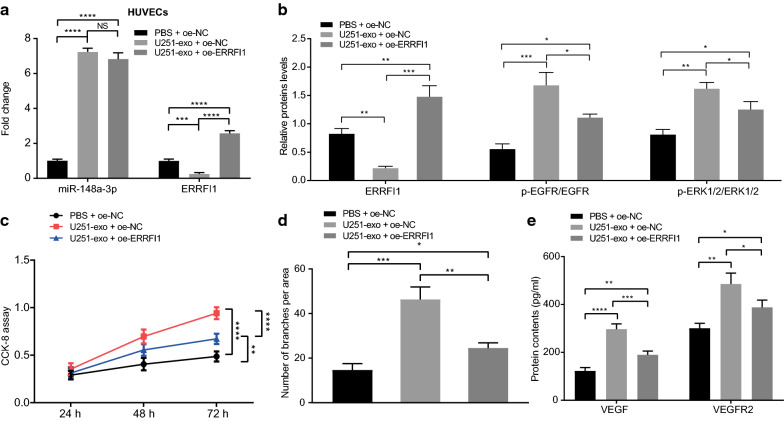
ERRFI1 expression is down-regulated by miR-148a-3p from U251 cells-derived exosomes to activate EGFR/MAPK signaling pathway, thereby enhancing tube formation ability of HUVECs. **a** Expression of miR-148a-3p and ERRFI1 in HUVECs was determined by RT-qPCR in the co-culture system of U251 cells-derived exosomes (U251-exo) and oe-NC/oe-ERRFI1-transiently transfected HUVECs (U6 and GAPDH were used as internal controls, respectively). **b** Western blot analysis of ERRFI1, ratios of p-EGFR/EGFR and p-ERK1/2/ERK1/2, and extent of ERK1/2/ERK1/2 phosphorylation in the co-culture system of U251-exo and oe-ERRFI1-transiently transfected HUVECs. **c** CCK-8 assay to evaluate the proliferation of oe-ERRFI1-transiently transfected HUVECs co-cultured with U251-exo. **d** Lumen formation ability of oe-ERRFI1-transiently transfected HUVECs co-cultured with U251-exo. **e** Expression of VEGF and VEGFR2 was tested using ELISA in cell supernatant in the co-culture system of oe-ERRFI1-transiently transfected HUVECs and U251-exo. * *p* < 0.05, ** *p* < 0.01, *** *p* < 0.001, and **** *p* < 0.0001 *vs.* the treatment with PBS + oe-NC or with U251-exo + oe-NC. U251 mentioned in this figure refers to U251-MG. Data are shown as mean ± SEM. The cell experiments were performed in 3 repeats and each repeat was performed in technical replicate (triplicate). Repeated measures ANOVA was used for data analysis among multiple groups at different time points. One-way ANOVA was used for multi-group comparison with Tukey's test

In order to examine how U251 cells-derived exosomes regulated the ERRFI1/EGFR/MAPK axis to influence HUVEC proliferation and angiogenesis, the exosomes isolated from U251 cells were co-cultured with HUVECs transfected with oe-ERRFI1 plasmid. RT-qPCR was then applied to analyze the expression patterns of miR-148a-3p and ERRFI1, which showed that U251 cells-derived exosomes could transfer the miR-148a-3p expression and inhibit the ERRFI1 expression in HUVECs, whereas transfection with oe-ERRFI1 rescued the ERRFI1 expression inhibited by U251 cells-derived exosomes (*p* < 0.001, *p* < 0.0001) (Fig. 5a). Consistently, Western blot analysis revealed that ERRFI1 protein expression was diminished, but the extent of EGFR and ERK1/2 phosphorylation was enhanced in the HUVECs when co-cultured with U251 cells-derived exosomes, both of which were noted to be counteracted by transfection with oe-ERRFI1 (*p* < 0.05, *p* < 0.01, *p* < 0.001) (Fig. 5b). CCK-8 and tube formation assays further revealed enhancements in HUVEC proliferation and angiogenesis after co-culture with U251 cells-derived exosomes, which were compromised by restoration of ERRFI1 (*p* < 0.05, *p* < 0.01, *p* < 0.001, *p* < 0.0001) (Fig. 5c, d). The contents of pro-angiogenic protein (VEGF and VEGFR2) in the cell supernatant detected by ELISA also demonstrated increases caused by U251 cells-derived exosomes, while restoration of ERRFI1 reversed these increases (*p* < 0.05, *p* < 0.01, *p* < 0.001, *p* < 0.0001) (Fig. 5e).

These data demonstrated that U251 cells-derived exosomes transferred miR-148a-3p to HUVECs to inhibit the expression of ERRFI1, which activated the EGFR/MAPK signaling pathway, leading to promoted HUVEC proliferation and angiogenesis.

### Exosomal miR-148a-3p induces tumor formation and angiogenesis in vivo

**Fig. 6:**
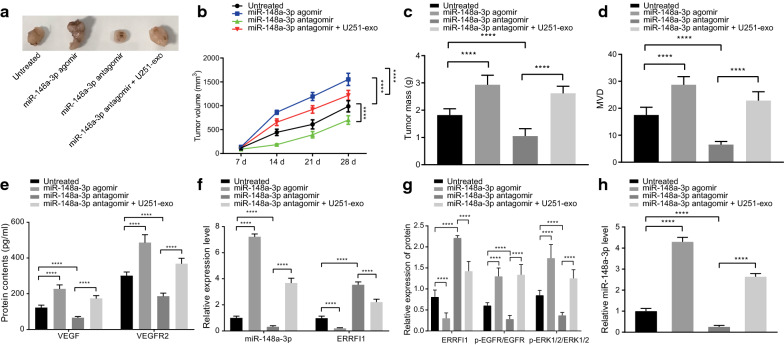
miR-148a-3p participates in the progression of glioma cell tumorigenesis and angiogenesis in vivo*.* The nude mice were injected with U251 cells to form xenografts and then injected with miR-148a-3p agomir, miR-148a-3p antagomir, or miR-148a-3p antagmir + U251-exo. **a** Representative images showing tumors formed in nude mice. **b** The volume of tumors in nude mice recorded every 7 days. **c** Tumor weight of nude mice. **d** IHC to quantify tumor MVD. **e** VEGF and VEGFR2 expression changes in nude mice detected by ELISA. **f** miR-148a-3p and ERRFI1 expressions were determined by RT-qPCR in tumor tissues of nude mice (U6 and GAPDH were used as internal controls, respectively). **g** Western blot analysis to detect ERRFI1, p-EGFR/EGFR, p-ERK1/2/ERK1/2 protein expressions in tumor tissues of nude mice. **h** Expression of miR-148a-3p was determined by RT-qPCR in serum exosomes in nude mice (cel-miR-39 was used as an internal reference). U251 mentioned in this figure refers to U251-MG. * *p* < 0.05, ** *p* < 0.01, *** *p* < 0.001, and **** *p* < 0.0001. Data are shown as mean ± SEM (n = 10). Repeated measures ANOVA was used for data analysis among multiple groups at different time points. One-way ANOVA was used for multi-group comparison with Tukey's test

Our preliminary findings revealed that miR-148a-3p in vitro can down-regulate the ERRFI1 expression, leading to the activation of the EGFR/MAPK signaling pathway, thereby enhancing proliferation and angiogenesis of HUVECs. In addition, in vivo experiments were performed to study the regulatory role of exosomal miR-148a-3p in the ERRFI1/EGFR/MAPK axis affecting tumor formation and angiogenesis in nude mice. Nude mice were injected subcutaneously with miR-148a-3p agomir, miR-148a-3p antagomir, or U251 cells-derived exosomes to alter the miR-148a-3p expression after U251 cell inoculation. The tumor growth curve of nude mice was plotted after monitoring for 4 consecutive weeks, and the results demonstrated that overexpression of miR-148a-3p by its agomir enhanced tumor growth (*p* < 0.0001) (Fig. 6a, b) and increased tumor weight (*p* < 0.0001) (Fig. 6c). In contrast, inhibiting miR-148a-3p by its antagomir in nude mice inhibited tumor growth (*p* < 0.0001) (Fig. 6a, b) and reduced tumor weight (*p* < 0.0001) (Fig. 6c). Furthermore, treatment of U251 cells-derived exosomes increased the tumor growth and weight by compromising the inhibitory effect of miR-148a-3p antagomir on miR-148a-3p (*p* < 0.0001) (Fig. 6a–c). These results demonstrated that exosomal transfer of miR-148a-3p promoted glioma tumor formation of in vivo.

Furthermore, results of IHC and ELISA showed that overexpression of miR-148a-3p by its agomir promoted tumor angiogenesis (CD31), and elevated the levels of VEGF and VEGFR2 in serum. However, miR-148a-3p inhibition by its antagomir suppressed tumor angiogenesis. Additionally, U251 cells-derived exosomes enhanced the miR-148a-3p expression and promoted tumor angiogenesis (CD31), in addition to elevating the serum levels of VEGF and VEGFR2 (*p* < 0.0001) (Fig. 6d, e). Meanwhile, the results of RT-qPCR and Western blot analysis showed that overexpression of miR-148a-3p by its agomir inhibited the ERRFI1 expression and activated the EGFR/MAPK signaling pathway; however, inhibition of miR-148a-3p by its antagomir up-regulated the ERRFI1 expression and blocked the EGFR/MAPK signaling pathway. In addition, it was found that U251 cells-derived exosomes enhanced the miR-148a-3p expression to inhibit ERRFI1 expression, and hence activate the EGFR/MAPK signaling pathway (*p* < 0.0001) (Fig. 6f, g). Finally, the expression of miR-148a-3p in the serum exosomes was analyzed by RT-qPCR, which illustrated that miR-148a-3p agomir treatment promoted the expression of miR-148a-3p in serum exosomes, while miR-148a-3p antagomir treatment inhibited the serum exosomal miR-148a-3p expression. Moreover, the treatment of U251 cells-derived exosomes increased the miR-148a-3p expression in serum exosomes (*p* < 0.0001) (Fig. 6h). In summary, our results demonstrated that up-regulation of miR-148a-3p by U251 cells-derived exosomes inhibited the ERRFI1 expression, so as to activate the EGFR/MAPK signaling pathway, thereby promoting glioma cell growth and angiogenesis in vivo.

## Discussion

Glioma is the most prevalent central nervous system neoplasm in adults characterized by poor prognosis [22]. Nowadays, routine therapies for glioma are surgery, chemotherapy, and radiation therapy; however, customized individually treatments that are based on dominant signaling pathways in tumor as well as tumor hallmarks have been cited to be more beneficial to glioma patients [23]. The poor prognoses of patients with glioma are possibly attributed to the high levels of angiogenesis [24]. In the current study, we set out to investigate the role of glioma cells-derived exosomal miR-148a-3p in the regulation of tumor angiogenesis. Collectively, our obtained findings revealed that that miR-148a-3p delivered by glioma cells-derived exosomes could promote tumor angiogenesis by activating the EGFR/MAPK signaling pathway through inhibition of the ERRFI1 expression.

Firstly, we uncovered that miR-148a-3p exhibited high expression in glioma tissues, cells and exosomes. Similar to our findings, up-regulated levels of miR-148a have also been previously recorded in glioblastoma tissues and cells [10]. In addition, another study documented aberrantly elevated levels of circulating exosomes-transported miR-148a in serum samples obtained from glioblastoma patients [8]. Furthermore, augmented expression of miR-148a has been documented in cell lines and stem cells of human glioblastoma, which further adds to the critical involvement of miR-148a in gliomas [19]. Meanwhile, a prior study has suggested that miR-148a also play a role in establishing a tumor microenvironment [25]. Moreover, Bai et al. found that exosome-transmitted miRNAs served as important mediators in the process of angiogenesis in gastric cancer, wherein malignant tumor cells would transmit information by means of exosomes, causing low-malignant tumor cells to attain high-malignant tumor cell characteristics and increased blood vessel formation [26], thus, we hypothesized a similar function of miR-148a-3p in gliomas. Our findings seemed to agree with the said hypothesis, revealing that glioma cells-derived exosomal miR-148a-3p exerted a promotive effect on the proliferation and angiogenesis of HUVECs, whereas inhibition of miR-148a-3p brought the opposite results. Consistent with our data, miR-148a-3p was reported to promote the expression of thrombospondin-4 to enhance endothelial cell angiogenesis in tendinopathy [27].

We also observed that glioma tissues, cells and exosomes presented with down-regulated levels of the ERRFI1 gene. Remarkably, ERRFI1 is lauded as a tumor suppressor gene of glioblastomas, whose high expression can diminish the migration of glioblastoma cells [14]. In addition, our findings further demonstrated that ERRFI1 was a target gene of miR-148a-3p, and could be directly targeted by miR-148a-3p. Interestingly, a previous study has shown that exosomes derived miR-126 possess the ability to regulate ERRFI1 to improve oxidative stress and apoptosis after the occurrence of ischemia and reperfusion injury [16]. Cai et al. proposed another mechanism stating that miR-148a could target cell adhesion molecule 1 (CADM1) gene to indirectly mediate the activity of STAT3, whereby promoting T98G cell growth and metastasis. However, this study only exhibited the enrichment of miR-148a in the exosomes derived from T98G cells and the negative correlation of CADM1 with exosomal miR-148a in patients with glioblastoma [8]. Furthermore, the current study incorporated the use of in vitro co-culture experimentation, and effectively identified the pro-angiogenic role of tumor exosomal miR-148a, which was further verified using in vivo tumor formation models. Nevertheless, these aforementioned findings from previous and present studies further enrich the important molecular mechanism of miR-148a in the progression of glioblastoma, providing a theoretical reference for the selection of clinical drug targets.

Another commonly noted feature of glioblastoma is the mutation and amplification of the EGFR gene [28]. The aberrant EGFR-mediated oncogenic signaling is found in glioblastoma [15]. Meanwhile, it has been reported that miR-130b can promote the progression of glioma by activating the ERK/MAPK signaling pathway [29]. Yang et al. [30] also demonstrated that silencing the AQP5 gene resulted in the inhibition of the EGFR/ERK/p38 MAPK signaling pathway, leading to repressed human glioma cell proliferation and migration, and augmented apoptosis. Our findings further demonstrated that miR-148a-3p could activate the EGFR/MAPK signaling pathway by targeting ERRFI1, wherein down-regulation of miR-148a-3p or up-regulation of ERRFI1 would suppress the EGFR/MAPK signaling pathway in HUVECs, leading to the inhibition of cell proliferation and angiogenesis. In line with our findings, low expression of miR-148a-3p has been previously shown to inhibit the progression of hepatocellular carcinoma with hepatitis C virus infection by suppressing c-Jun and the MAPK signaling pathway [31].

## Conclusion

Taken together, findings obtained in the current study demonstrate that glioma cells-derived exosomal miR-148a-3p activate the EGFR/MAPK signaling pathway by repressing the expression of the ERRFI1 gene, leading to the promotion of tumor angiogenesis in glioma. Our work sheds a new light on the underlying mechanism behind miR-148a-3p functioning in glioma, and also highlights its value serving as novel therapeutic target for glioma treatment. However, other roles of miR-148a-3p in the development and prognosis of glioma warrant further investigation.

## Supplementary information

**Additional file 1: Table S1.** Clinical characteristics of glioma patients and the inclusion criteria. **Table S2.** The STR profiling of HEK-293T cell line, HUVEC cell line and U138-MG, U251-MG and LN229 cell lines.

**Additional file 2.** The original Western Blot images.

## Data Availability

The datasets generated/analyzed during the current study are available.

## Abbreviations

miRNAs or miRs: MicroRNAs
ERRFI1: ERBB receptor feedback inhibitor 1
HUVECs: Human umbilical vein endothelial cells
EGFR: Epidermal growth factor receptor
MAPK: Mitogen-activated protein kinase
DNMT1: DNA methyltransferase-1
GEO: Gene Expression Omnibus
STR: Short tandem repeats
FBS: Fetal bovine serum
TEM: Transmission electron microscope
ECL: Enhanced chemiluminescence
FDR: False discovery rate
HA: Human astrocytes
ER: Endoplasmic reticulum
CANX: Calnexin
VEGF: Vascular endothelial growth factor
LSCM: Laser scanning confocal microscope
RPMI: Roswell Park Memorial Institute
RT-qPCR: Reverse transcription-quantitative polymerase chain reaction
oe: Overexpression
NC: Negative control
SDS-PAGE: Sodium dodecyl sulfate polyacrylamide gel electrophoresis
cDNA: Complementary DNA
FBS: Fetal bovine serum
ELISA: Enzyme-linked immunosorbent assay
OD: Optical density
CCK-8: Cell counting kit-8
MVD: Microvascular density
GAPDH: Glyceraldehyde-3-phosphate dehydrogenase
PBS: Phosphate-buffered saline
DMEM: Dulbecco's modified Eagle's medium
PBST: PBS containing 0.5% Tween-20
DAPI: 4′,6-Diamidino-2-phenylindole
WT: Wild type
MUT: Mutant
3′UTR: 3′-Untranslated region
RLU: Relative luciferase units
ANOVA: Analysis of variance
CADM1: Cell adhesion molecule 1
