# Supplementary material for: Glioma exosomal microRNA-148a-3p promotes tumor angiogenesis through activating the EGFR/MAPK signaling pathway via inhibiting ERRFI1
Source: Cancer Cell Int. 2020 Oct 27;20:518. doi: 10.1186/s12935-020-01566-4 (PMC7590612; doi:10.1186/s12935-020-01566-4)
Supplement: Supplementary file 1 — Additional file 1: Table S1. Clinical characteristics of glioma patients and the inclusion criteria. Table S2. The STR profiling of HEK-293T cell line, HUVEC cell line and U138-MG, U251-MG and LN229 cell lines. [file 12935_2020_1566_MOESM1_ESM.docx]

**Table S1.** Clinical characteristics of glioma patients and the inclusion criteria

|  | Patients underwent intracranial decompression surgery | Glioma patients |
| --- | --- | --- |
| Sample size | n = 20 | n = 45 |
| Gender | 12 males and 8 females | 27 males and 18 females |
| Age (years) | 50.10 ± 14.33 | 48.09 ± 11.06 |
| Tumor location |  | frontal lobe (n = 12) |
|  |  | temporal lobe (n = 22) |
|  |  | occipital lobe (n = 11) |
| Tumor typing |  | astrocytoma (n = 7) |
|  |  | oligodendroglioma (n = 4) |
|  |  | anaplastic astrocytoma (n = 9) |
|  |  | anaplastic oligodendroglioma (n = 6) |
|  |  | glioblastoma (n = 19) |
| Tumor classification |  | WHO I-II grade (n = 24) |
|  |  | WHO III-IV grade (n = 21) |
| Inclusion criteria | patients with hypertensive intracerebral hemorrhage were required to receive "intracranial decompression surgery”;  patients had the normal brain tissues in non-functional areas could be taken during the operation | patients pathologically diagnosed with glioma;  patients had no treatment for glioma in the past 3 months;  patients with complete clinical data |

**Table S2.** The STR profiling of HEK-293T cell line, HUVEC cell line and U138-MG, U251-MG and LN229 cell lines

| Cell line | AMEL | D5S818 | D13S317 | D7S820 | D16S539 | vWA | THO1 | TPOX | CSF1PO |
| --- | --- | --- | --- | --- | --- | --- | --- | --- | --- |
| HEK-293T | X | 18,9 | 12,14 | 11 | 9,13 | 16,19 | 7,9.3 | 11 | 11,12 |
| HUVEC | X | 11,12 | 9,11 | 8,12 | 11,12 | 16 | 6,9.3 | 8,11 | 11,12 |
| U-138MG | XY | 11 | 9,11 | 9 | 12,13 | 18 | 6 | 8 | 12 |
| U-251 MG | XY | 11,12 | 10,11 | 10,12 | 12 | 16,18 | 9.3 | 8 | 11,12 |
| LN229 | X | 11,12 | 10,11 | 8,11 | 12 | 16,19 | 9.3 | 8 | 12 |

Note: STR, short tandem repeat; HUVEC,human umbilical vein endothelial cell.
