# Supplementary figures and images for: Glioma exosomal microRNA-148a-3p promotes tumor angiogenesis through activating the EGFR/MAPK signaling pathway via inhibiting ERRFI1
Source: Cancer Cell Int. 2020 Oct 27;20:518. doi: 10.1186/s12935-020-01566-4 (PMC7590612; doi:10.1186/s12935-020-01566-4)

fig1C


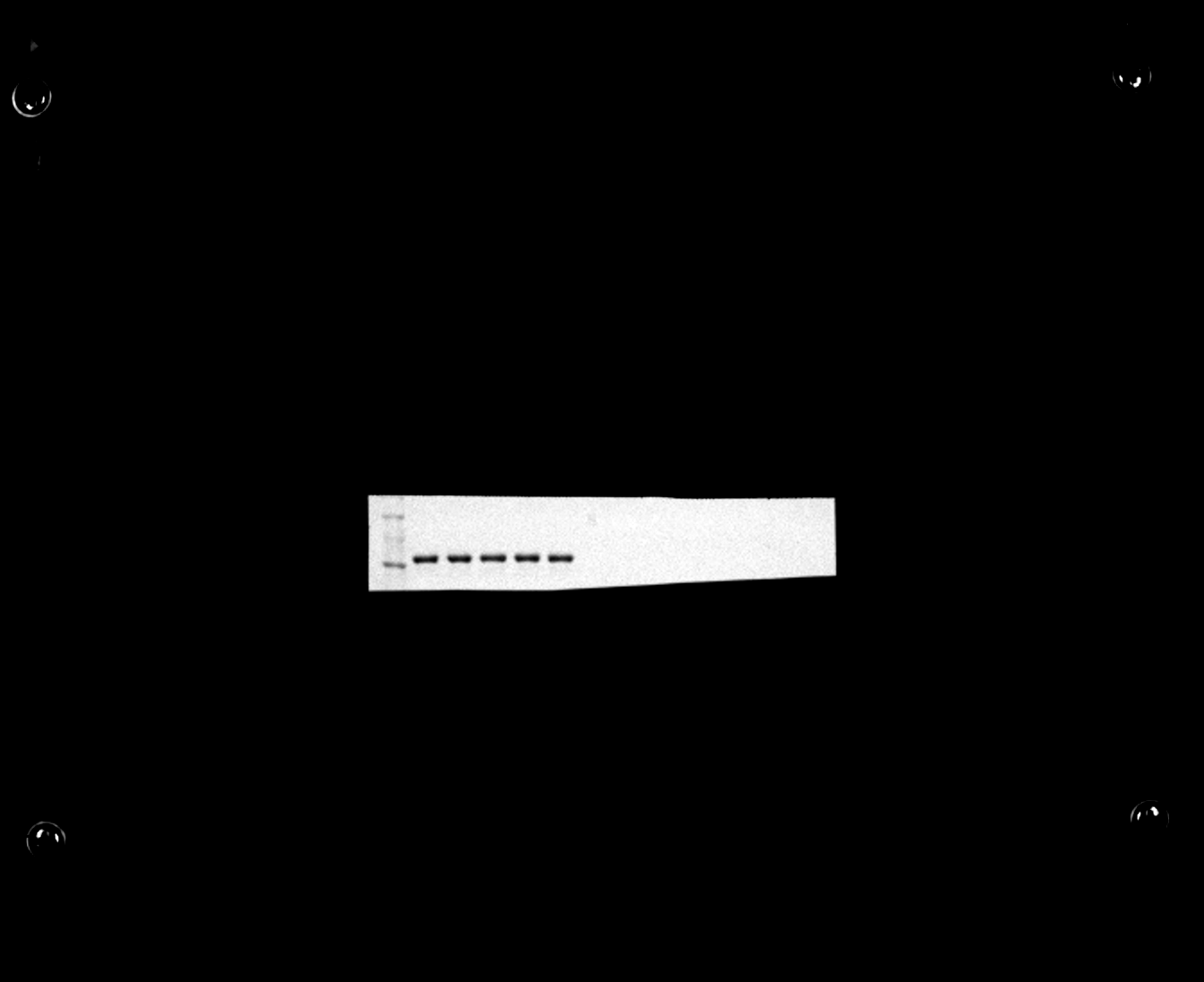


CANX


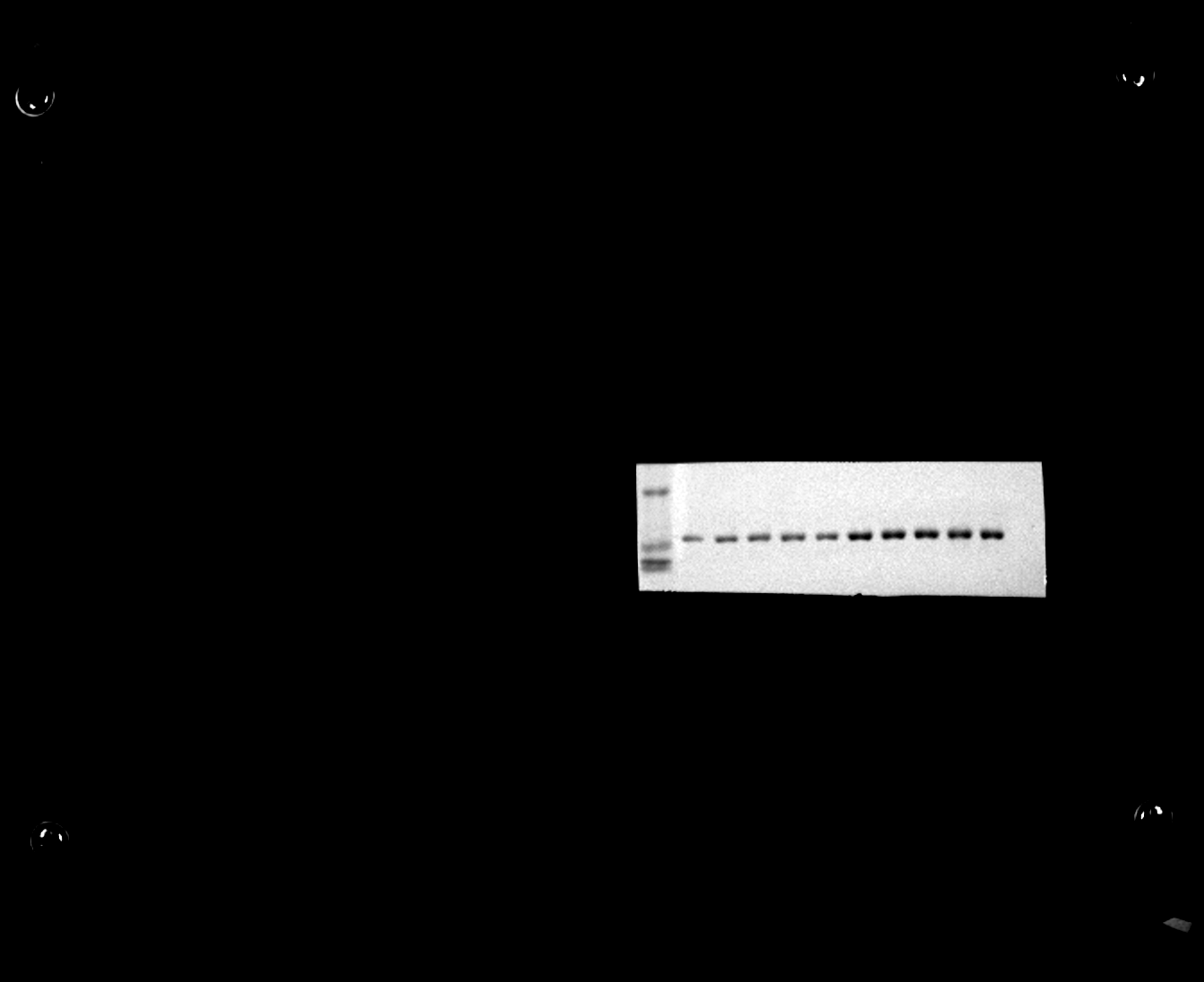


CD63


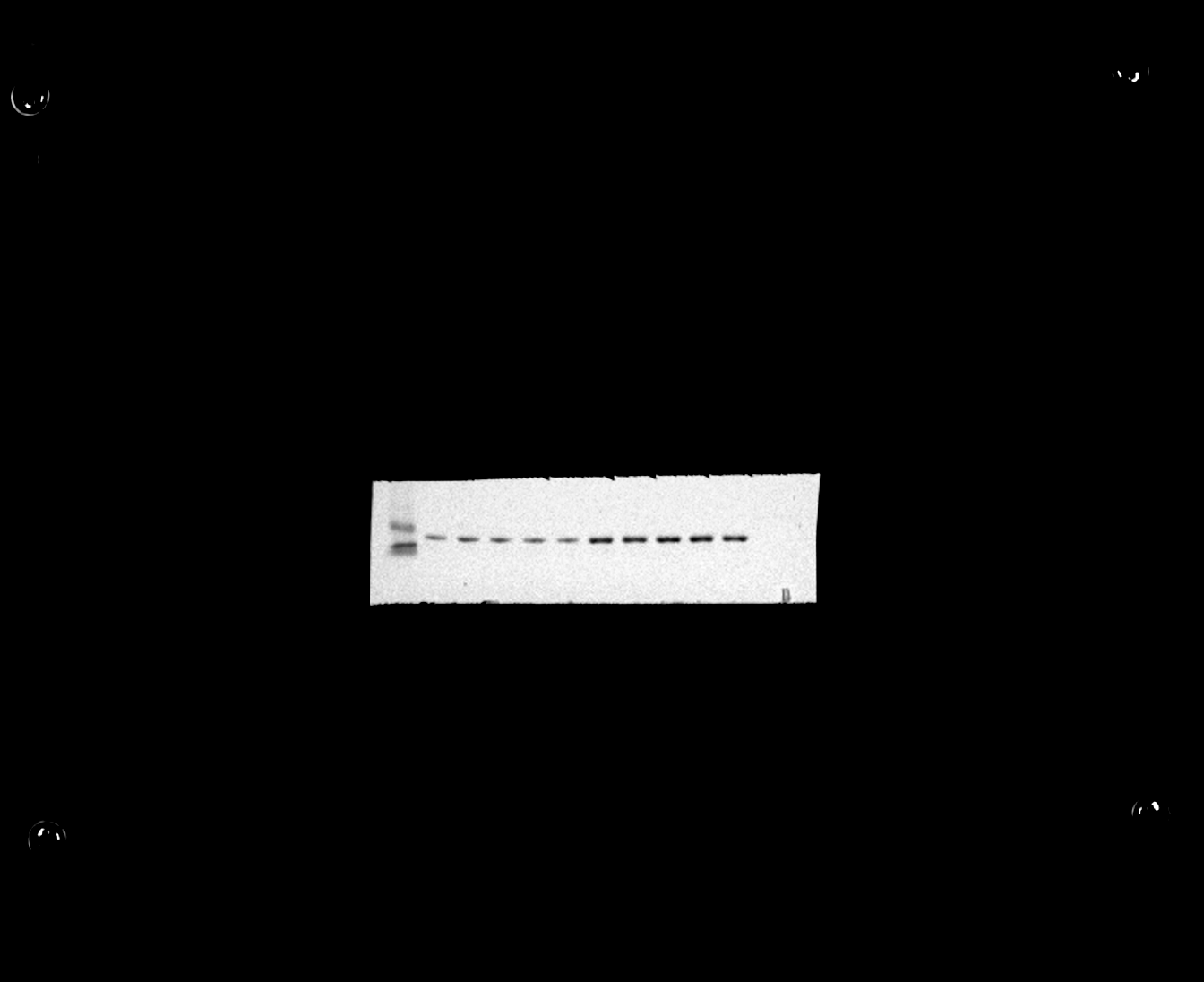


CD81


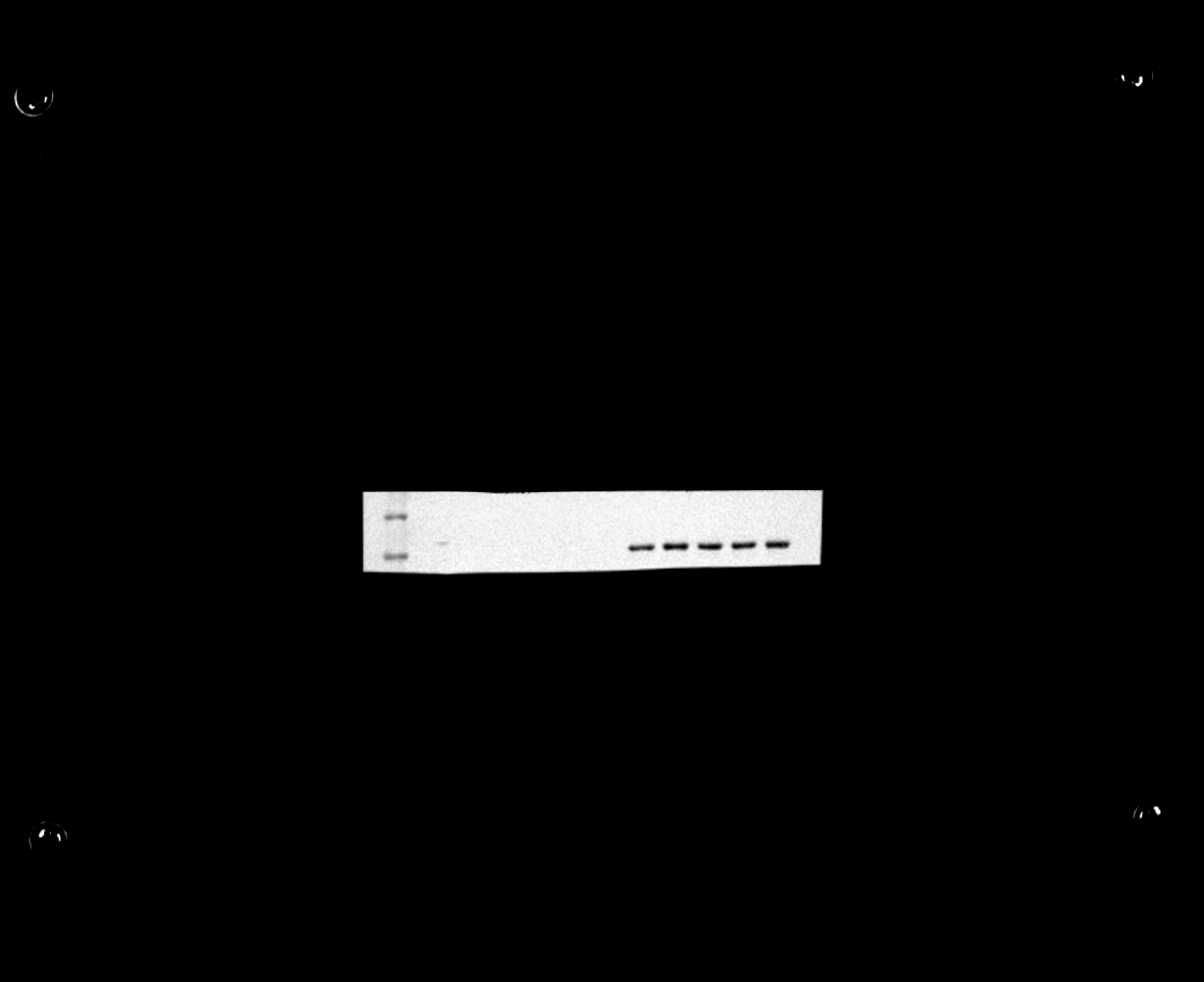


TSG101

fig4F


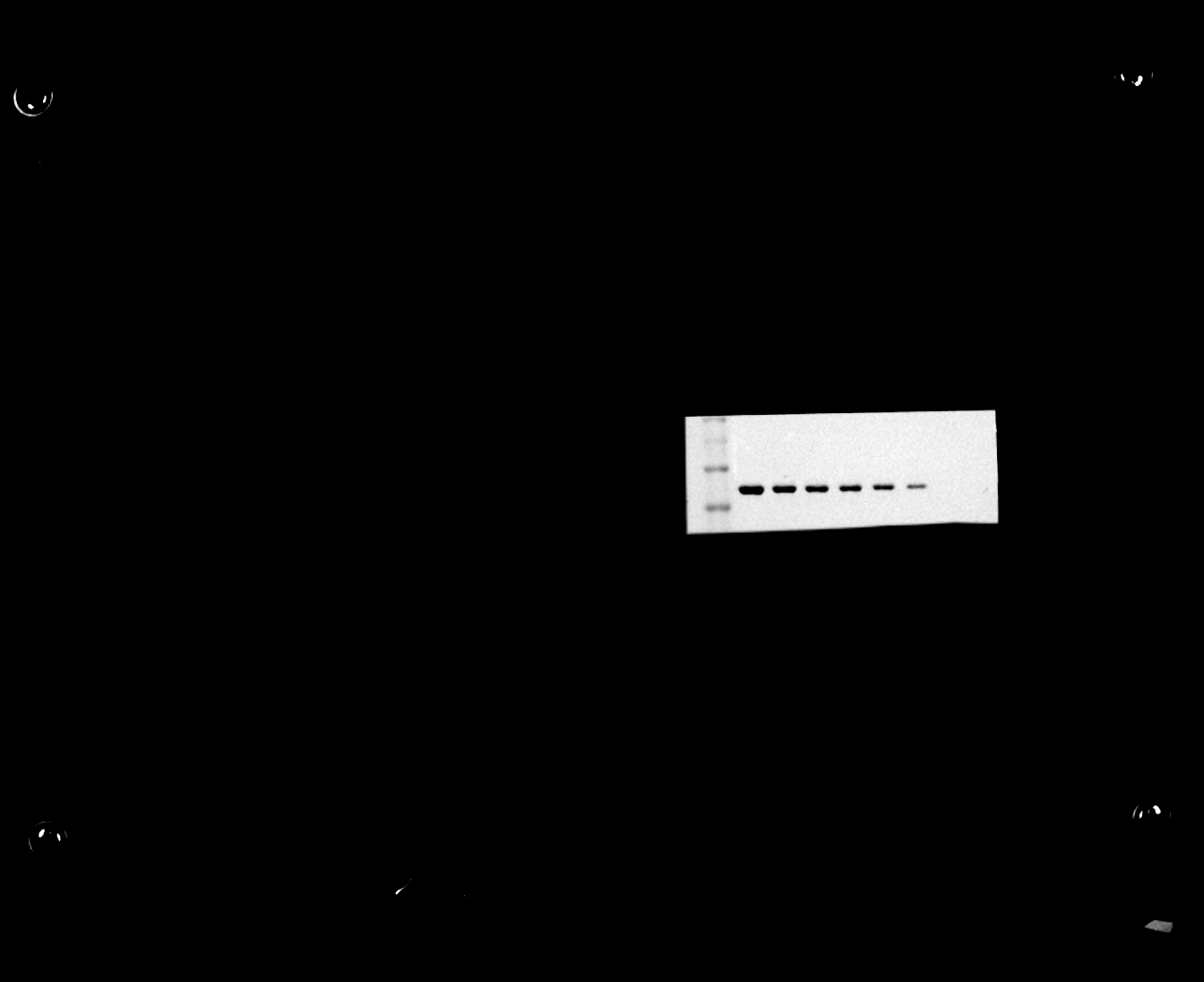


ERRFI1


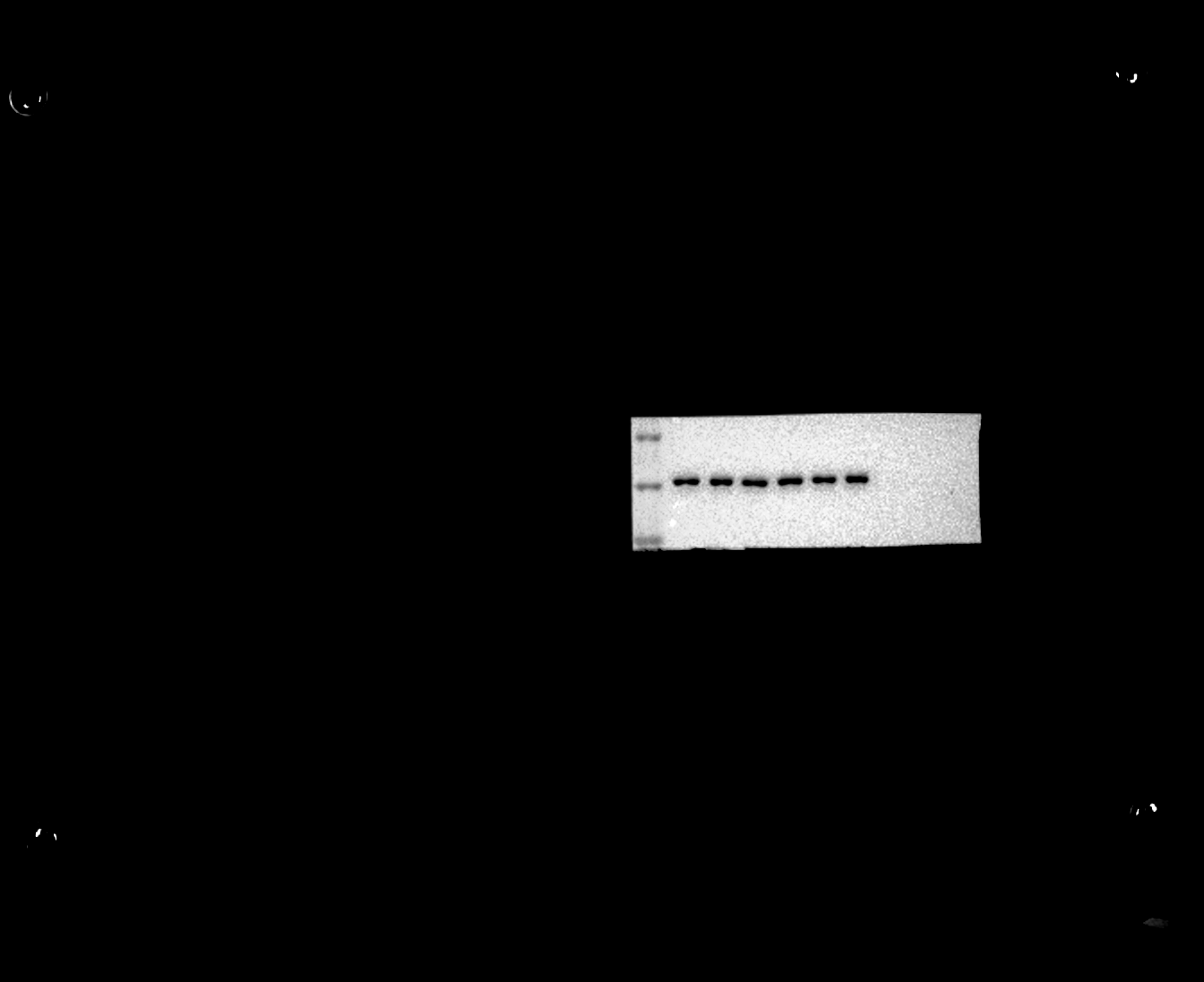


GAPDH

fig4I


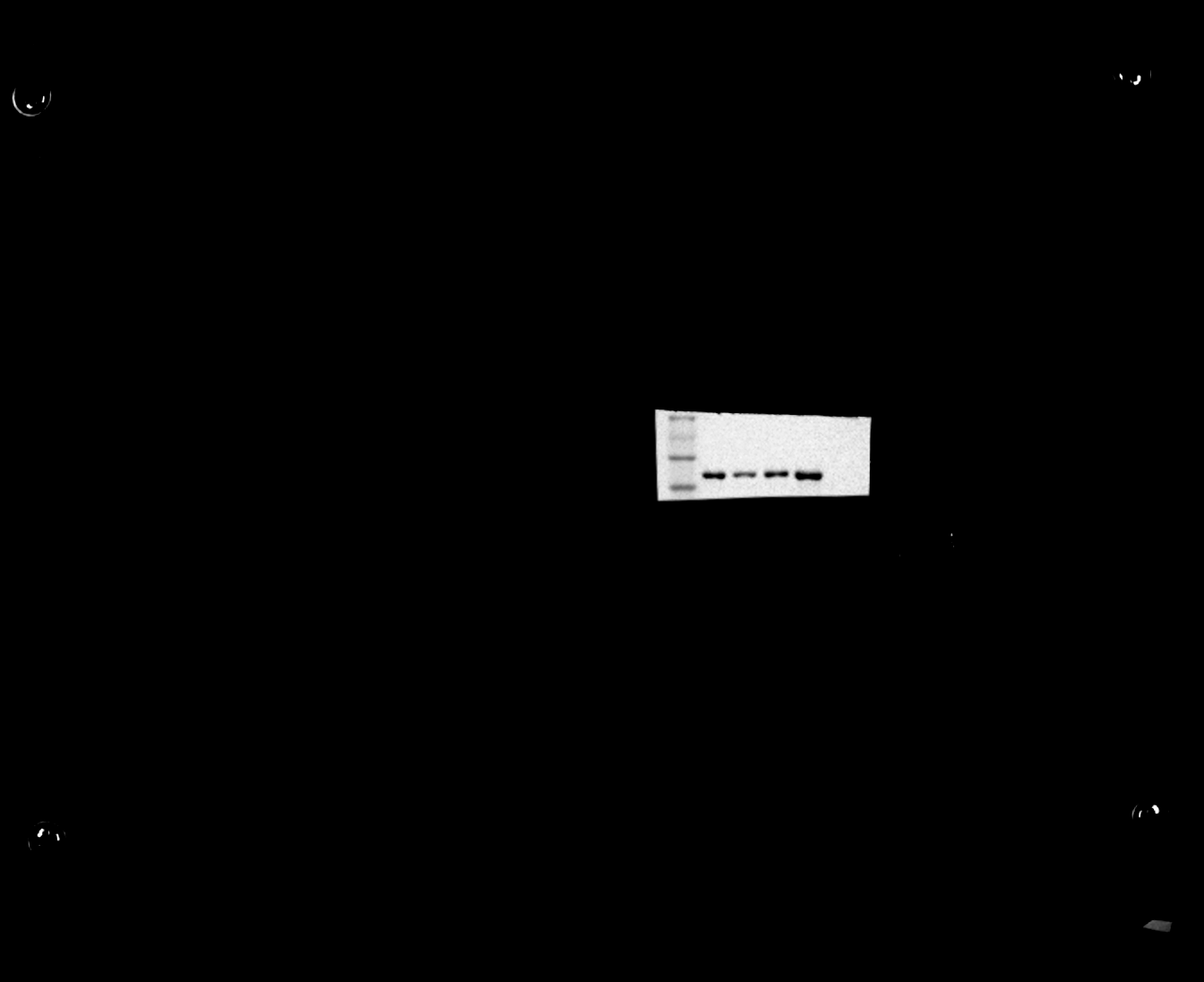


ERRFI1


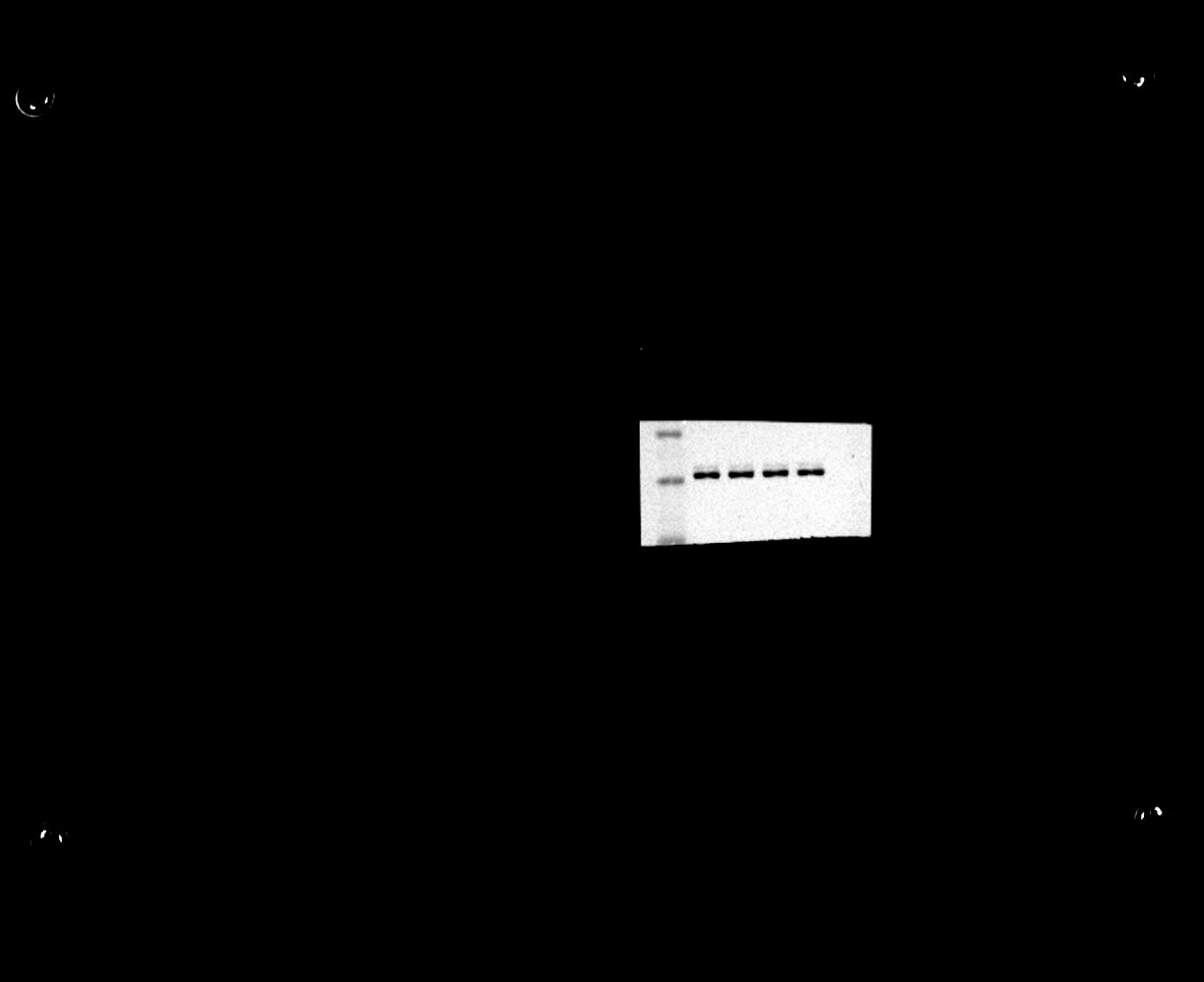


GAPDH

Supplement: Supplementary file 2 — Additional file 2. The original Western Blot images. [file 12935_2020_1566_MOESM2_ESM.docx]
